# The biodiversity of ice‐free Antarctica database

**DOI:** 10.1002/ecy.70000

**Published:** 2025-01-27

**Authors:** Aleks Terauds, Jasmine R. Lee, Hannah S. Wauchope, Ben Raymond, Dana M. Bergstrom, Peter Convey, Claire Mason, Charlotte R. Patterson, Sharon A. Robinson, Anton Van de Putte, David Watts, Steven L. Chown

**Affiliations:** ^1^ Integrated Digital East Antarctica Program Australian Antarctic Division, Department of Climate Change, Energy, the Environment and Water Kingston Tasmania Australia; ^2^ Securing Antarctica's Environmental Future Queensland University of Technology Brisbane Queensland Australia; ^3^ British Antarctic Survey, NERC Cambridge UK; ^4^ School of GeoSciences University of Edinburgh Edinburgh UK; ^5^ School of Earth, Atmospheric and Life Sciences University of Wollongong Wollongong New South Wales Australia; ^6^ Department of Zoology University of Johannesburg Auckland Park South Africa; ^7^ Millennium Institute ‘Biodiversity of Antarctic and Sub‐Antarctic Ecosystems’ (BASE) Santiago Chile; ^8^ Cape Horn International Center (CHIC) Puerto Williams Chile; ^9^ CSIRO Environment Hobart Tasmania Australia; ^10^ Institute for Marine and Antarctic Studies University of Tasmania Hobart Tasmania Australia; ^11^ Centre for Data Science Queensland University of Technology Brisbane Queensland Australia; ^12^ Securing Antarctica's Environmental Future University of Wollongong Wollongong New South Wales Australia; ^13^ Biodiversity and Ecosystems Data and Information Centre Royal Belgian Institute of Natural Sciences Brussels Belgium; ^14^ Marine Biology Lab Université Libre de Bruxelles Brussels Belgium; ^15^ CSIRO National Collections and Marine Infrastructure Information and Data Centre Hobart Tasmania Australia; ^16^ Securing Antarctica's Environmental Future, School of Biological Sciences Monash University Melbourne Victoria Australia

**Keywords:** Antarctica, arthropods, biogeography, ice‐free, lichens, moss, penguins, seabirds, terrestrial biodiversity

## Abstract

Antarctica is one of Earth's most untouched, inhospitable, and poorly known regions. Although knowledge of its biodiversity has increased over recent decades, a diverse, wide‐ranging, and spatially explicit compilation of the biodiversity that inhabits Antarctica's permanently ice‐free areas is unavailable. This absence hinders both Antarctic biodiversity research and the integration of Antarctica in global biodiversity–related studies. Fundamental and applied research on biodiversity patterns, ecological structure and function, and options for conservation are reliant on spatially resolved, taxonomically consistent observations. Such information is especially important for modern, data‐driven biodiversity science, in both Antarctica and globally, and forms the backbone of biodiversity informatics, reflected, for example, in the Darwin Core Standard used by the Global Biodiversity Information Facility. Biodiversity data are also essential to fulfill the conservation requirements for Antarctica, as set out in the Protocol on Environmental Protection to the Antarctic Treaty and inform the design of systematic surveys to address biodiversity and ecological knowledge gaps, for both specific taxa and ecosystems. Such surveys are key requirements for understanding and mitigating the impacts of environmental change on the region's biodiversity. Here, we address these requirements through the public release of The Biodiversity of Ice‐free Antarctica Database. In 2008, we extracted a subset of biodiversity records only from terrestrial ice‐free areas from the Scientific Committee on Antarctic Research (SCAR) Antarctic Biodiversity Database. We have subsequently added thousands of records from a range of sources: checking, and where necessary (and possible), correcting the spatial location, clarifying, cross‐referencing, and harmonizing taxonomy with globally recognized sources, and documenting the original source of records. The Biodiversity of Ice‐free Antarctica Database spans the early 1800s to 2019 (with most records collected after 1950) and represents the most comprehensive consolidation of Antarctic ice‐free biodiversity occurrence data yet compiled into a single database. The Biodiversity of Ice‐free Antarctica Database contains 35,654 records of 1890 species in over 800 genera across six kingdoms and spans all Antarctic Conservation Biogeographic Regions. These data are released under a CC BY Attribution License (http://creativecommons.org/licenses/by/4.0/).

## CONFLICT OF INTEREST STATEMENT

The authors declare no conflicts of interest.

## Supporting information


Data S1.

Metadata S1.

## Data Availability

Data are available as Supporting Information (Data S1) and are also archived through the Australian Antarctic Data Centre at https://doi.org/10.4225/15/59100ba9157f7.

