# Supplementary material for: The biodiversity of ice‐free Antarctica database
Source: Ecology. 2025 Jan 27;106(1):e70000. doi: 10.1002/ecy.70000 (PMC11772906; doi:10.1002/ecy.70000)
Supplement: Supplementary file 2 — Metadata S1. [file ECY-106-e70000-s002.docx]

## Data Paper published in *Ecology*, an Ecological Society of America journal

## Metadata S1

**The biodiversity of ice-free Antarctica database**

Aleks Terauds^1,2^, Jasmine R. Lee^2,3^, Hannah S. Wauchope^4^, Ben Raymond^1^, Dana M. Bergstrom^1,5^, Peter Convey^3,6,7,8^, Claire Mason^9,10^, Charlotte R. Patterson^2,11^, Sharon A. Robinson^5,12^, Anton Van de Putte^13,14^ David Watts^15^ and Steven L. Chown^16^

^1^ Integrated Digital East Antarctic Program, Australian Antarctic Division, Department of Climate Change, the Environment, Energy and Water, Kingston, Tasmania, Australia.

^2^ Securing Antarctica’s Environmental Future, Queensland University of Technology, Brisbane, QLD, Australia

^3^ British Antarctic Survey, NERC, High Cross, Madingley Road, Cambridge CB3 0ET, UK

^4^ School of GeoSciences, University of Edinburgh, Edinburgh, United Kingdom

^5^ School of Earth, Atmospheric & Life Sciences, University of Wollongong, Wollongong, NSW, Australia

^6^ Department of Zoology, University of Johannesburg, Auckland Park, South Africa

^7^ Millennium Institute 'Biodiversity of Antarctic and sub-Antarctic Ecosystems' (BASE), Santiago, Chile

^8^ Cape Horn International Center (CHIC), Puerto Williams, Chile

^9^ CSIRO Environment, Hobart, Tasmania, Australia

^10^ Institute for Marine and Antarctic Studies, University of Tasmania, Hobart, Tasmania, Australia

^11^ Centre for Data Science, Queensland University of Technology, Brisbane, Queensland, Australia

^12^ Securing Antarctica’s Environmental Future, University of Wollongong, Wollongong, NSW, Australia

^13^ Biodiversity and Ecosystems Data and Information Centre, Royal Belgian Institute of Natural Sciences, Vautierstraat 29 1000, Brussels Belgium

^14^ Marine Biology Lab,Université Libre de Bruxelles, Franklin Roosevelt 50, 1050 Brussels Belgium

^15^ CSIRO National Collections and Marine Infrastructure Information and Data Centre, Hobart Tasmania

^16^ Securing Antarctica’s Environmental Future, School of Biological Sciences, Monash University, Melbourne, Victoria 3800, Australia

Corresponding Author: Aleks Terauds. Email: [Aleks.Terauds@aad.gov.au](mailto:Aleks.Terauds@aad.gov.au)

**Open Research Statement:** Data are available as Supporting Information (Data S1) and are also archived through the Australian Antarctic Data Centre at <https://doi.org/10.4225/15/59100ba9157f7>.

# **Introduction**

Continental Antarctica remains one of the most remote, inhospitable, and least explored regions of the planet (e.g., Leihy et al., 2020). Collective knowledge of nature and the spatial distribution of biodiversity across ice-free areas of continental Antarctica has increased substantially over recent decades but is still patchy, and in some areas, non-existent (Terauds et al., 2012; Terauds & Lee, 2016; Hawes et al., 2023). Despite this lack of knowledge, the global importance and uniqueness of Antarctic biodiversity is well recognized (Chown et al., 2015; Lee et al., 2022), and the quest to better understand Antarctic biota has been a fundamental driver of Antarctic research since the earliest days of exploration (e.g., the BANZARE and Discovery Expeditions; Deacon, 1955; Mawson, 1932).

Most Antarctic biodiversity is found in the permanently ice-free areas (Lee et al., 2017), and is often described as species-poor compared to other regions of the globe. However, life in the continent’s ice-free areas is structurally and functionally diverse and present in all but the most severe habitats (Chown et al., 2015; Convey and Peck, 2019; Dragone et al., 2021; Ortiz et al., 2021). This biodiversity includes moss, lichens, fungi, invertebrates (e.g., mites, springtails, nematodes, rotifers, tardigrades), microbes, and breeding birds and seals (Shirihai, 2008; Terauds et al., 2012; Convey et al., 2014; Chown et al., 2015).

The biogeography of ice-free Antarctica has been of interest for decades (Gressitt, 1961; Chown & Convey, 2007; Terauds et al., 2012; Terauds & Lee., 2016; Araujo et al., 2020; Convey et al., 2020). Clarifying the spatial structure and environmental drivers of Antarctic biodiversity has also been the subject of numerous studies (see Convey et al., 2014; Chown et al., 2015; Lee et al., 2019, and references therein), as has clarifying the risks and impacts of non-native species (Chown et al., 2012; Duffy et al., 2017; Hughes et al., 2020), and understanding the functional diversity of a range of organisms (e.g., Chan et al., 2013; Chown et al., 2015). All of these studies required knowledge of species occurrences, however, all of them have acknowledged data gaps and patchiness as a limitation. The paucity of consolidated Antarctic biodiversity data is also likely to be a key reason for the absence of Antarctica across a range of global studies (e.g., Pacifici et al., 2015; Hoskins et al., 2020)

These data limitations highlight that one of the most fundamental requirements for biodiversity research and conservation is not being met for terrestrial Antarctica. Biodiversity data are essential to fulfil the conservation requirements for Antarctica, as set out in the Protocol on Environmental Protection to the Antarctic Treaty (<https://www.ats.aq/e/protocol.html>). Designing systematic surveys to address biodiversity knowledge gaps is also more difficult when little to no information are available for both specific taxa and particular geographic locations, such as is the case for much of Antarctica. Such surveys are key requirements for understanding ecological structure and function, and mitigating the impacts of environmental change on the region’s biodiversity.

The Scientific Committee on Antarctic Research (SCAR), an affiliated body of the International Science Council (ISC) and the primary scientific body for coordinating international Antarctic research (Hughes et al., 2023), has played an important role in the collation and dissemination of Antarctic biodiversity knowledge and has been instrumental in providing support for several of the above studies. One of the best examples is the SCAR Antarctic Biodiversity Database (AADC, 2002), which was initiated and compiled through the SCAR Regional Sensitivity to Climate Change (RiSCC) and Evolution and Biodiversity in Antarctica (EBA) Programs, which operated from 2000 – 2013. The SCAR Antarctic Biodiversity Database continues to be hosted and maintained by the Australian Antarctic Data Centre (AADC, 2002). No records have been added to the SCAR Antarctic Biodiversity Database since 2008, but SCAR continues to encourage initiatives to facilitate the compilation and dissemination of biodiversity information, including the database presented here (see also Acknowledgements).

Here we present *The Biodiversity of Ice-free Antarctic Database* – a consolidation of spatially explicit occurrence records from a wide range of sources. This database represents the most comprehensive consolidation of terrestrial occurrence records at a continental scale yet compiled into a single database, and will underpin future regional and global studies of ecology, diversity and change.

The process of compiling this database began in 2008, when we extracted a subset from the SCAR Antarctic Biodiversity Database focusing on records **only from ice-free areas** (Table 1). This subset formed the foundation of the database presented here (and subsequently underpinned the first delineation of the Antarctic Conservation Biogeographic Regions - ACBRS - Terauds et al., 2012). We built upon this foundation between 2008 and 2023 by undertaking systematic searches of the Antarctic literature on the occurrence of species in ice-free areas, adding thousands of records from published sources, other databases and grey literature. As part of the data quality-assurance quality-control process we removed spurious records and duplicates, checked, and where necessary, corrected the spatial location, cross-referenced and harmonized taxonomy with globally recognized sources, and wherever possible, documented the original source of records. While some records still lack complete consensus on final taxonomy, it was not possible to resolve all of these and, wherever possible, we used globally recognized sources. We also ensured, wherever possible, that taxonomy was harmonised between this database and the SCAR Antarctic Biodiversity Database (AADC, 2002).

The formatting of this database is consistent with Darwin Core Standards to maximise the sharing use and reuse of these data. It is also consistent with recent calls made for improved consolidation and accessibility of biological data, both for Antarctica (O’Brien et al., 2022) and globally (McCrea et al., 2023). The database presented here will continue to change with time to reflect contemporary understanding of biodiversity in Antarctic ice-free area.

Iterations of the database presented have supported a range of studies since 2008, including continental biogeography (Terauds et al., 2012; Terauds & Lee, 2016); protection of biodiversity (Wauchope et al., 2019), an assessment of wilderness (Leihy et al., 2020) and an Antarctic species inventory (Pertierra et al., 2024).

*The Biodiversity of Ice-free Antarctic Database* contains 35,654 records of 1,890 species in over 800 genera across six kingdoms (Fig. 1, Fig. 2). Records span all Antarctic Conservation Biogeographic Regions (Fig. 3), with over three-quarters found within 1 km of the Antarctic coastline (Figure 4). The occurrence records were collected between the early 1800s and 2019, with most collected after 1950 (Figure 5). A comprehensive assessment of the geographic, environmental, taxonomic, and temporal coverage of the database presented here is provided in a companion paper (Patterson et al., *in press*), which highlights potential strengths and limitations and discusses use of these data in further analysis, including species distribution modelling.


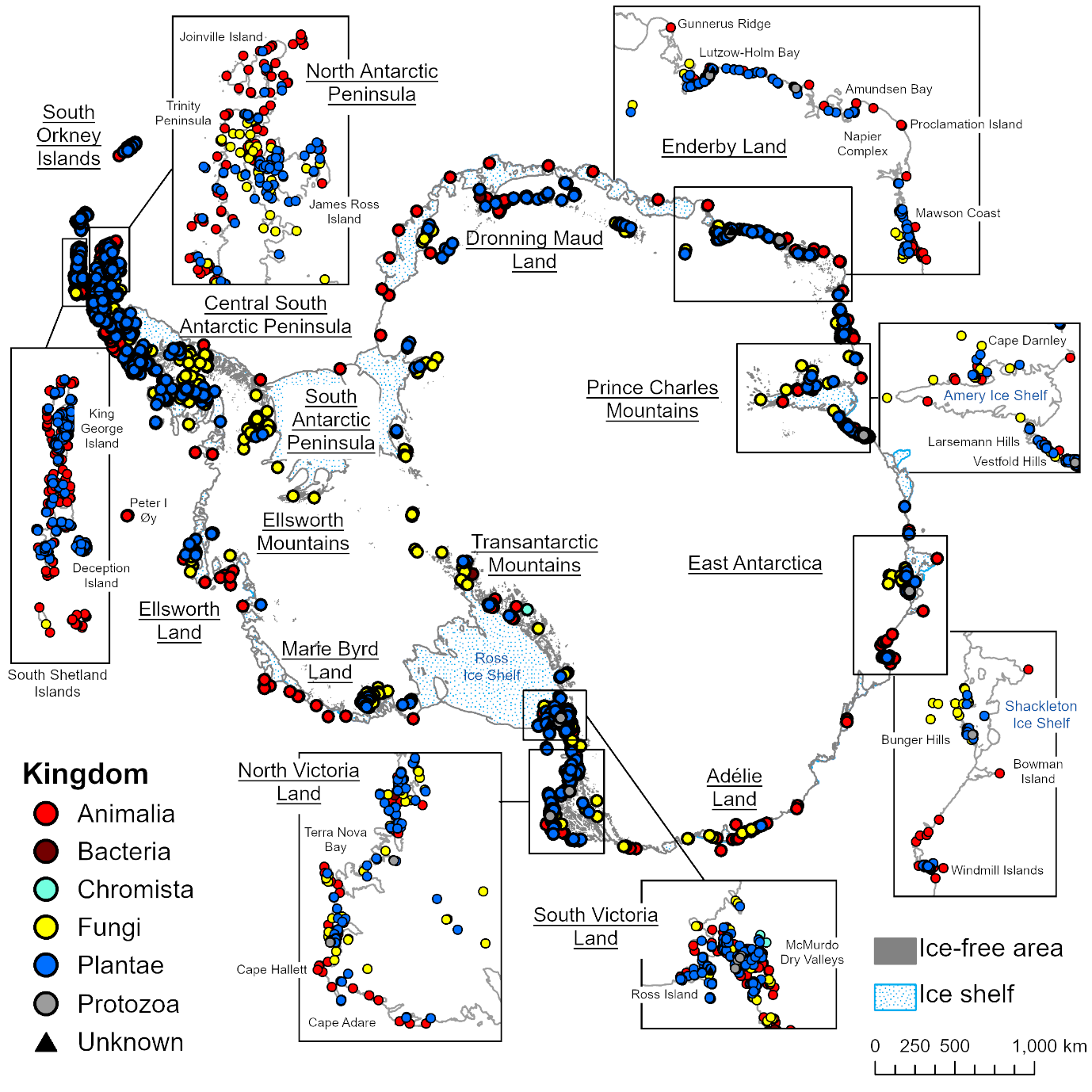


**Figure 1.** Continent wide occurrence records (>35,000) from *The Biodiversity of Ice-free Antarctica Database*, coloured by taxonomic kingdom. Records were collected between the early 1800s and 2019, with most collected after 1950. Names of Antarctic Conservation Biogeographic Regions (ACBRs) are underlined (‘North-east’ and ‘North-west’ Antarctic Peninsula combined). Insets are provided for the North Antarctic Peninsula, South Shetland Islands, North and South Victoria Land, Enderby Land, the Prince Charles Mountains, and parts of the East Antarctic.


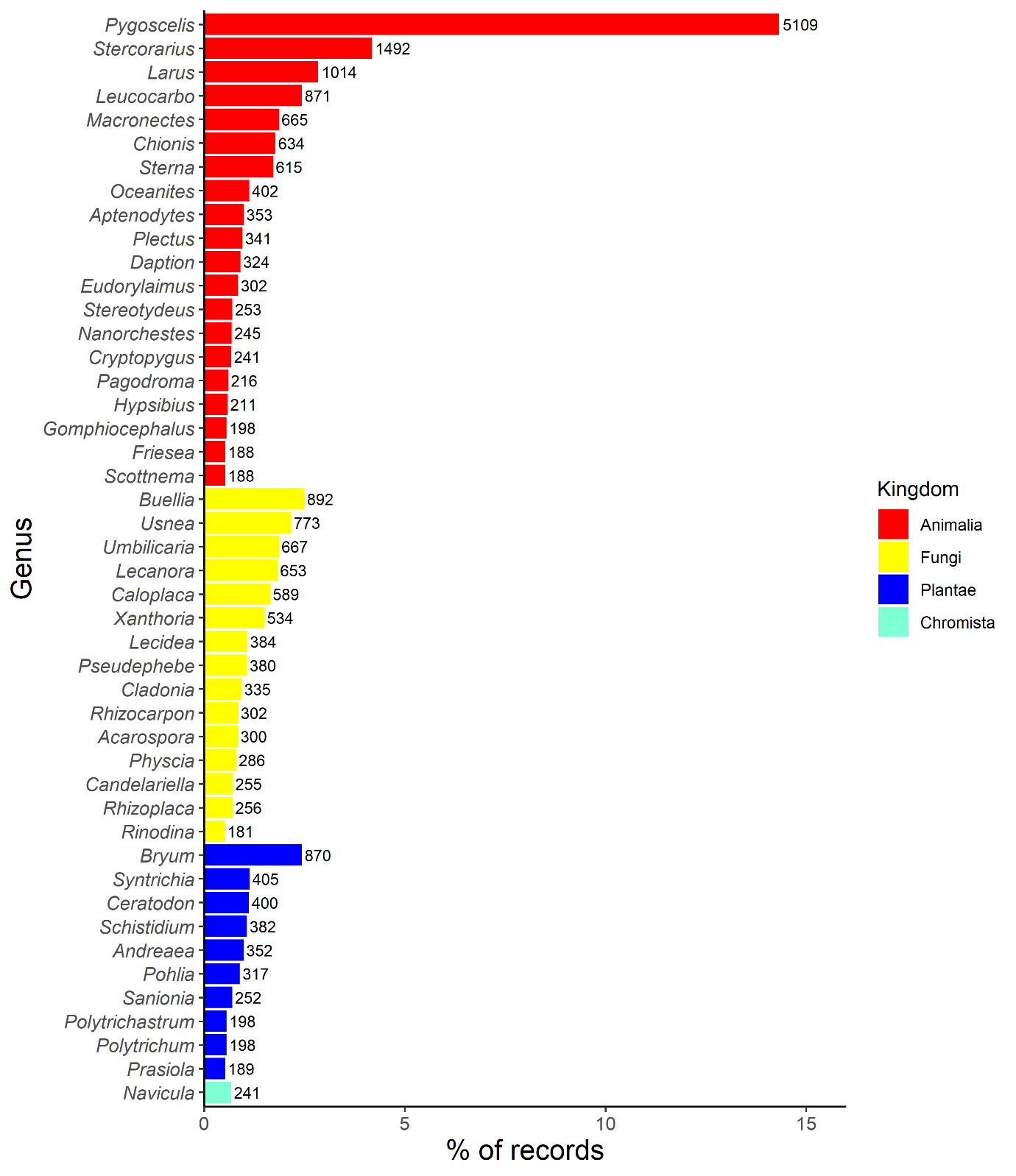


**Figure 2.** The percentage of records in *The Biodiversity of Ice-free Antarctica Database* by genus (number of records next to bar). Only genera with >0.5% of records are included, whilst non-included genera make up ~30% of records in total. Colours represent taxonomic kingdom and match the colours in Figure 1.


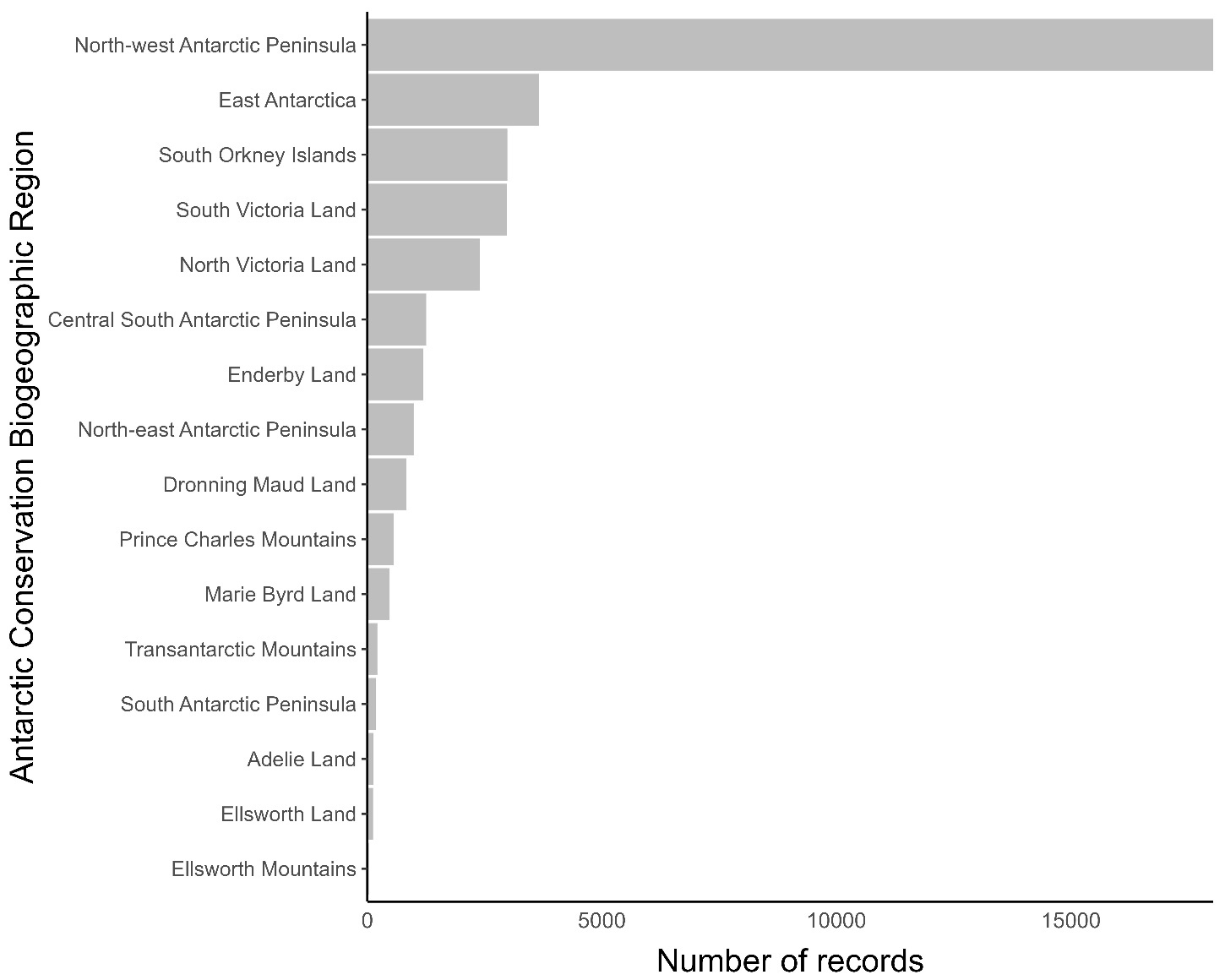


**Figure 3.** The number of *Ice-free Antarctic Biodiversity Database* records that occur in each of the Antarctic Conservation Biogeographic Regions (see Figure 1 for locations of ACBRs).


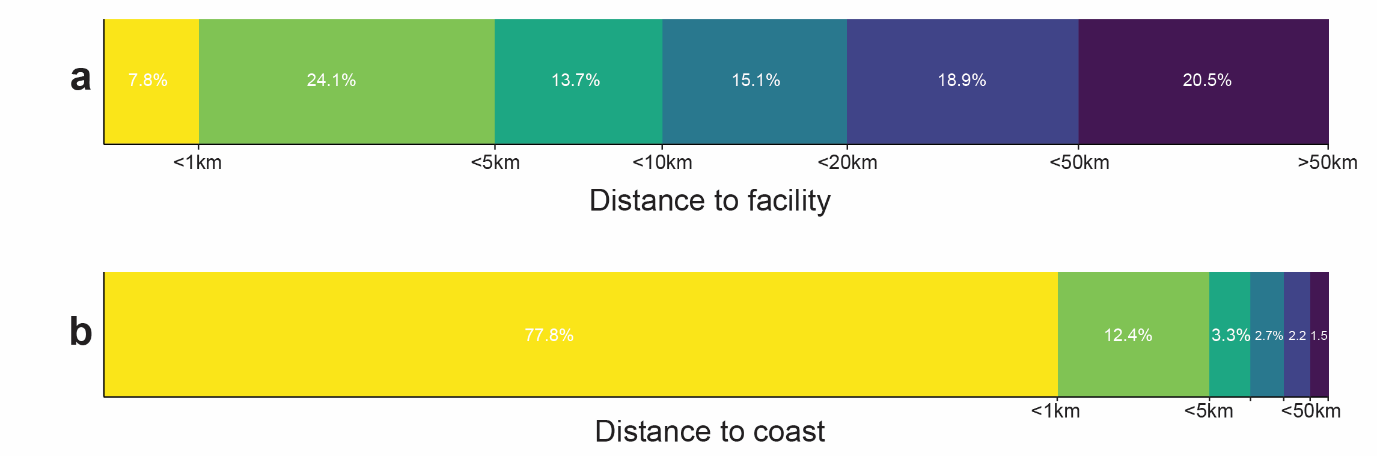


**Figure 4.** The percentage of records in distance classes from a) Antarctic facilities (i.e. permanent infrastructure) and b) the nearest coastline.


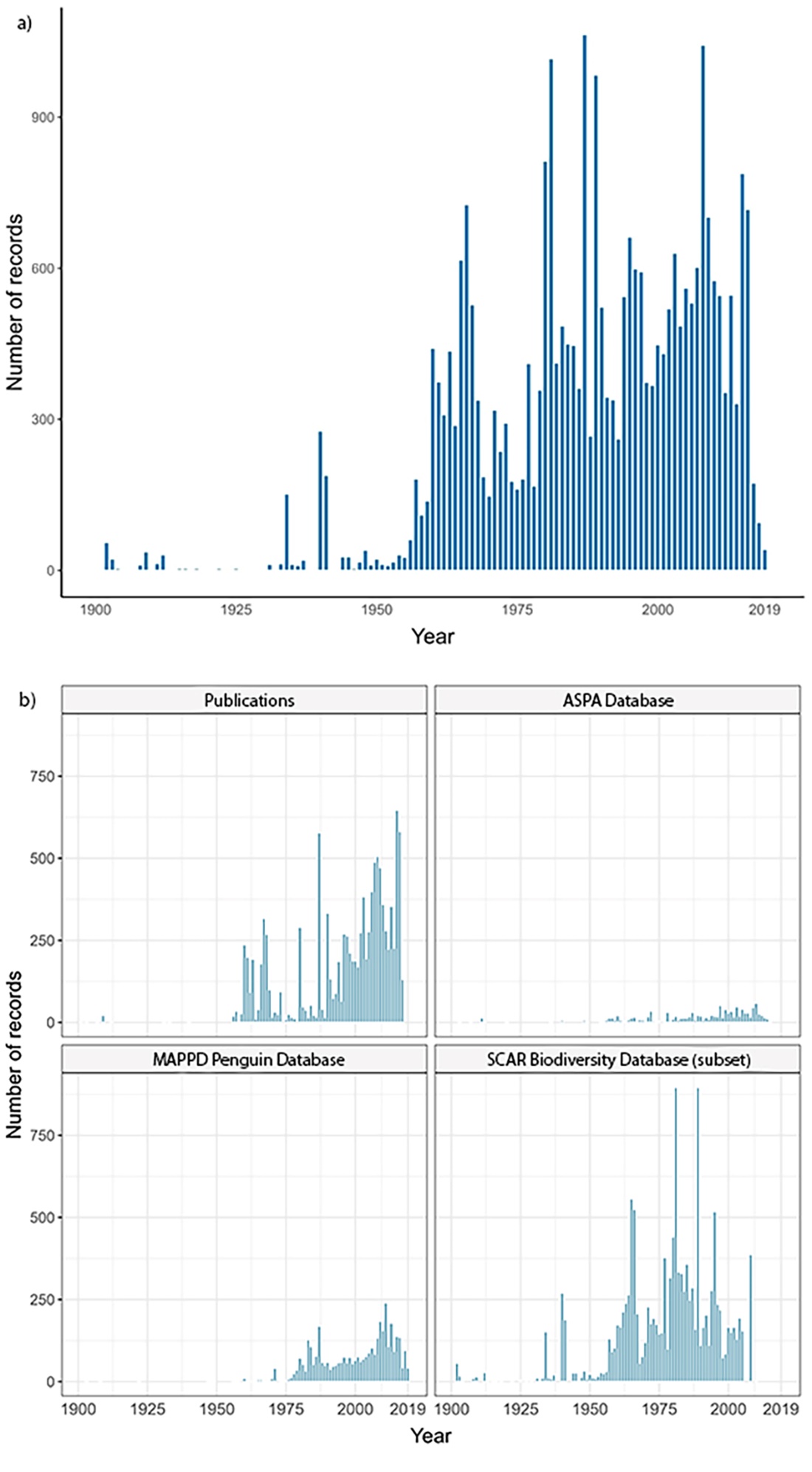


**Figure 5.** Frequency histogram of the date of collection for records that had temporal data (excluding the <10 records prior to 1900) for a) all data and b) by source (also see Table 3).

| Collection Name | Area | Number of records | Number of unique taxa | Main taxa | Custodian |
| --- | --- | --- | --- | --- | --- |
| Australian Antarctic Division Herbarium | Antarctica | 3919 | 132 | Moss, liverworts, lichens, vascular plants | Rod Seppelt |
| BAS Antarctic Plant Database (to 2004) | Antarctica | 23096 | 929 | Fungi, liverworts, moss, lichens, vascular plants | Peter Convey, Helen Peat |
| John Gibson invertebrate collection | Antarctica | 406 | 162 | Arthropods, nematodes, mites, tardigrades, fleas | John Gibson |
| Cape Hallett Springtails | Cape Hallett | 9 | 5 | Collembola | Brent J. Sinclair |
| BAS invertebrate collection | Antarctica | 2672 | 90 | All Antarctic invertebrates | Helen Peat |
| Nematodes | Antarctica | 65 | 18 | Nematodes | Istvan Andrassy |
| NIPR Bryophyte Specimens Database | Antarctica | 2837 | 46 | Mosses and liverworts | Satoshi Imura |
| Argentine Island moss and lichens | Argentine Island | 697 | 68 | Mosses and lichens | Niek J.M. Gremmen |
| Northern Victoria Land Lichens (PNRA Database) | Northern Victoria Land | 1094 | 42 | Lichens | Miris Castello,  Stefano Martellos |
| Antarctic Algae database | Antarctica | 3368 | 570 | Terrestrial and freshwater algae | Bruno Fumanti,  P. Cavacini |
| AAD Electron Micrographs | Antarctica | 305 | 51 | Lichens and mosses | Geraldine Nash |
| Nematodes | Antarctica | 65 | 18 |  | Istvan Andrassy |
| Moss from Windmill Is. and Vestfold Hills | Windmill Island and Vestfold Hills | 408 | 2 | Moss | Sharon A. Robinson |

**Table 1.** A summary of the original data collections extracted in 2008 from the SCAR Antarctic Biodiversity Database (AADC, 2002), which were used as the foundation for *The Biodiversity of Ice-free Antarctica Database* presented here.

## ­Class I. Data Set Descriptors

1. **Data set identity:**

The Biodiversity of Ice-free Antarctica Database

1. **Data set identification codes:**

Biodiversity_IceFree_Antarctica.csv

1. **Data set description**
   - - 1. **Principal Investigators:**

**Aleks Terauds,** Integrated Digital East Antarctica Program, Australian Antarctic Division, Department of Climate Change, Energy, the Environment and Water and Environment, Kingston, 7050, Tasmania, Australia; and, Securing Antarctica’s Environmental Future Queensland University of Technology, Brisbane, QLD, Australia

**Steven L. Chown,** Securing Antarctica’s Environmental Future, School of Biological Sciences, Monash University, Melbourne, Victoria 3800, Australia

**Jasmine R. Lee,** British Antarctic Survey, NERC, High Cross, Madingley Road, Cambridge CB3 0ET, UK

- - - 1. **Abstract:**

Antarctica is one of Earth’s most untouched, inhospitable, and poorly known regions. Although knowledge of its biodiversity has increased over recent decades, a diverse, wide-ranging, spatially explicit compilation of the biodiversity that inhabits Antarctica’s permanently ice-free areas is unavailable. Fundamental and applied research on biodiversity patterns, ecological structure and function, and options for conservation are reliant on spatially resolved, taxonomically consistent observations. Such information is especially important for modern, data-driven biodiversity science, and forms the backbone of biodiversity informatics, reflected for example in the Darwin Core Standard used by the Global Biodiversity Information Facility. Biodiversity data are also essential to fulfil the conservation requirements for Antarctica, as set out in the Protocol on Environmental Protection to the Antarctic Treaty and inform the design of systematic surveys to address biodiversity and ecological knowledge gaps, for both specific taxa and ecosystems. Such surveys are key requirements for understanding and mitigating the impacts of environmental change on the region’s biodiversity. Here, we address these requirements through the public release of *The Biodiversity of Ice-free Antarctica Database*. In 2008, we extracted a subset of biodiversity records only from terrestrial ice-free areas, from the Scientific Committee on Antarctic Research (SCAR) Antarctic Biodiversity Database. We have subsequently added thousands of records from a range of sources; checking, and where necessary (and possible), correcting the spatial location, clarifying, cross-referencing and harmonizing taxonomy with globally recognized sources, and documenting the original source of records. This database represents the most comprehensive compilation of Antarctic ice-free biodiversity occurrence data yet compiled into a single database and spans the early 1800s to 2019, with most collected after 1950. The database contains 35,654 records of 1,890 species in over 800 genera across six kingdoms and spans all Antarctic Conservation Biogeographic Regions.

1. **Key words/phrases:**

Antarctica; Arthropods; Biogeography; Ice-free; Lichens; Moss; Penguins; Seabirds; Terrestrial Biodiversity

## Class II. Research origin descriptors

1. **Overall project description:**
   1. **Identity:**

Compilation of biodiversity occurrence data from Antarctic ice-free areas.

- 1. **Originators:**

The database presented here was built on a subset of the SCAR Antarctic Biodiversity Database (AADC, 2002). The subset was extracted in 2008 and no further records have been added to the SCAR Antarctic Biodiversity Database since.

The SCAR Antarctic Biodiversity Database was initiated and compiled through the SCAR Regional Sensitivity to Climate Change (RiSCC) and Evolution and Biodiversity in Antarctica (EBA) Programs. The focus on ice-free biodiversity and extraction of the ice-free subset was initiated by Steven Chown, Peter Convey, Dana Bergstrom, and Aleks Terauds in 2008 (see Table 1 for the original data sources and their custodians), with the actual data extraction facilitated by David Watts of the Australian Antarctic Data Centre. Several researchers have since contributed substantially to the compilation and cross-checking of the database presented here, and have been included as co-authors to this Data Paper. Most recently, Jasmine Lee has led the finalisation of the database and, together with Aleks Terauds, led the writing of the Metadata.

- 1. **Period of study:**

The occurrence records span from the early 1800s to 2019 (Figure 5). Adding records, taxonomic harmonization, cross-checking, and validation of data were undertaken between 2008 and 2023 – see Class II B 2 and Class II B 3 for further details. Table 2 provides a more detailed breakdown of the date of observation/collection of the occurrence records.

- 1. **Objectives:**

To compile extensive and comprehensive occurrence data from ice-free areas in Antarctica. To ensure that the spatial location was as accurate as possible and that the taxonomy was harmonised with recognized sources where possible (e.g. Catalogue of Life, Global Biodiversity Information Facility, World Register of Marine Species – see Class II B 3 Taxonomic harmonisation for further details) .

- 1. **Abstract:**

See Class I. C. 2. Abstract

- 1. **Sources of funding:**

The Scientific Committee for Antarctic Research has provided significant support for this work, largely through its Research Programs, including Regional Sensitivity to Climate Change (RiSCC), Evolution and Biodiversity in Antarctica (EBA), The State of the Antarctic Ecosystem (AntEco) and most recently through the Integrated Science to Support Antarctic and Southern Ocean Conservation (Ant-ICON). The Australian Antarctic Science Program provided support through the Integrated Digital East Antarctic Program (IDEA) and grants: AAS 4296 (Aleks Terauds) and AAS 4297 (Jasmine Lee). The Australian Research Council supported this research through SRIEAS Grant SR200100005 Securing Antarctica’s Environmental Future. Jasmine Lee was also supported by a Royal Commission for the Exhibition of 1851 Research Fellowship. Peter Convey is supported by NERC core funding to the BAS ‘Biodiversity, Evolution and Adaptation’ Team. The Belgian Science Policy office provided support (EU-LifeWatch contract n° FR/36/ AN1/AntaBIS, ADVANCE contract n° RT/23/ADVANCE).

1. **Specific subproject description**
   1. **Site description:**

We classified ice-free biodiversity as any species or equivalent taxon that occurs on one or more of the permanently ice-free areas of Antarctica during any part of its life cycle. Given the boundary between ice-free areas and limnetic life and ice-free areas and intertidal life in Antarctica is not always clearly defined, where there was uncertainty, species were retained. For example, penguins and seals that breed or spend time on ice-free areas were included, as were migratory birds like skuas. Microbe occurrences were included when they came from the extract from the SCAR Antarctic Biodiversity Database, or reported as part of a biological survey, but typically data from eDNA and sequenced Operational Taxonomical Units (OTUs) were not included.

Biodiversity records were collated from all continental ice-free areas and the Antarctic Peninsula islands, its associated islands, South Orkney Islands, Peter I Island, and the Balleny Islands. The actual spatial coverage is best represented by the Antarctic Conservation Biogeographic Regions (Terauds & Lee, 2016; shapefile available at <http://dx.doi.org/10.4225/15/5729930925224>), which was based on the Medium Resolution Rock Outcrop Layer of the Antarctic Digital Database (V 7.1 Gerrish et al., 2020).

Recent estimates of the overall extent of ice-free areas range from 0.2% of the Antarctic continent (21,745km^2^ – Burton-Johnson et al., 2016) to 0.4% (54,274km^2^ – Brooks et al., 2019). These ice-free areas are patchily distributed through a landscape dominated by snow and ice and can occur in a range of forms, including coastal oases, cliffs, nunataks and scree (Convey et al., 2018; Lee et al., 2022). Their relatively small size, patchy distribution and isolation from each other have led to these habitats being described as ‘islands in the ice’ (Lee et al., 2022). Several reviews provide overviews of terrestrial (ice-free) Antarctic biodiversity (Chown et al., 2015), its biogeographical distribution (Chown & Convey, 2007; Terauds et al., 2012; Terauds & Lee, 2016; Convey et al., 2020), the environmental context (Convey et al., 2018; Brooks et al., 2019; Leihy et al., 2020), climate-related changes (Lee et al., 2017), environmental drivers (Convey et al., 2014) and its vulnerability to a range of threatening anthropogenic processes (Convey & Peck., 2019; Lee et al., 2022 a,b).

- 1. **Data Collection**

**Starting Point:** 38,876 occurrence records from 13 collections were extracted from the Antarctic Biodiversity Database (Table 1) in June 2008. No information on the collection date of these records was available at the time of extraction and no absence data, and no count data, were available from these collections. An initial assessment of the extracted data was made to ensure they a) were spatially explicit, b) were within the spatial scope (i.e., in ice-free areas), c) only included species that occurred on ice-free areas (as defined above). Records that did not meet these criteria were deleted (see Class II B 3 below for more details).

**Adding Records**: Records were added from a range of published sources (between 2009 and 2019), which were identified using a systematic search of the published literature through Web of Science and Google Scholar. Search terms ranged from very broad (e.g., “Antarctic* and “bio*) to more specific (e.g., “Antarctic* AND nematode AND survey”). Searches were iterative and adaptive, changing and often increasing in complexity and specificity to reflect the knowledge gained from previous searches. Hundreds of combinations of search terms were used between 2009 and 2019 in order to track down as many relevant studies as possible. The title of a paper was used as the first indicator of relevance, followed by the Abstract, Methods and Results. In many cases, the bibliographies of relevant papers were used to find further studies with spatially explicit occurrence data.

Once a paper was found with spatially explicit information on the occurrence of a species (i.e., a coordinate or place name) the paper was saved in a library, with its bibliographic information saved in a separate file. Occurrence data from the paper were entered into a separate spreadsheet and cross referenced to that paper. At the culmination of the search process (i.e. once all the relevant papers had been entered), we used the *melt* function in the *reshape* package (Wickham et al., 2007) in R (R Core Team, 2019) to consolidate these data into a table with a new row for each occurrence record. Any records that were not specific to at least genus were excluded, as were records that were uncertain at a generic level (records uncertain to species were listed as 'Genus sp.').

Other sources of occurrence records included a publicly available online database of penguin colony locations (penguinmap.com) and the ASPA Biodiversity Database (Wauchope et al., 2019), which was generated from records in Management Plans of Antarctic Specially Protected Areas - <https://www.ats.aq/devph/en/apa-database> ). Table 3 provides a summary of the main source categories of biodiversity occurrence data.

Where the only spatial data was a place name, we first attempted to retrieve coordinates from the SCAR Composite Gazetteer (<https://data.aad.gov.au/aadc/gaz/scar/>). In the rare event the place name was not included in the SCAR Gazetteer, the information from the original source was used to identify the location in Google Earth and extract coordinates. Location accuracy was further assessed by checking in Google Earth or other internet sources (and where possible recorded in the coordinateUncertaintyInMetres field – Table 4).

Finally, we went through and corrected the species names for typographic and spelling errors and allocated a Taxon ID (provided by the Australian Antarctic Data Centre). Further checks were made of all records during the database finalisation process. This included the consolidation of synonyms (see Class II Section B 3 below for further details).

- 1. **Research methods**

**Data checking, deletion and consolidation:** Only spatially explicit records were included in the database. The following process was applied to data from the original 13 collections and new records that were added.

1. If only a place name was provided, the SCAR Gazetteer - (https://data.aad.gov.au/aadc/gaz/scar/) was used to allocate a latitude and longitude.
2. Each record was then checked to see if it overlapped with Antarctic Digital Database rock outcrop layer (Version 7.1 medium resolution - <https://www.scar.org/resources/antarctic-digital-database/> ).
3. Where records did not overlap (i.e., they appeared to be on ice or at sea) and were confirmed as taxonomically in scope (see above), they were checked to see if they were within the broad spatial scope (Class II B1) and if outside this spatially defined area they were deleted.
4. If records appeared to be within the spatial scope and were within 20 km of an ice-free area, they were moved to the nearest ice-free area, excluding some seabird colonies nesting on offshore islands (that were not represented in the ice-free layer). 20 km was chosen as a realistic yet conservative representation of the potential error for lower resolution coordinates.
5. If records were >20 km away from an ice-free area, the location was double checked with the SCAR Gazetteer. Again, if it was confirmed that the record did not occur on ice-free land, the record was deleted.
6. Details of original location and distance moved were retained, and an estimate of the spatial accuracy was provided where possible (Table 4).

**Taxonomic harmonisation:** Taxonomic classifications of species records were checked to ensure that *a)* classifications were up-to-date, and *b)* that species synonyms were resolved wherever possible. The workflow can be summarised as:

1. The taxonomic classification of all species listed in the database were first checked against the Catalogue of Life(COL; Bánki et al., 2024; <https://www.catalogueoflife.org/data/browse>) and corrected as necessary.
2. Genera absent from the COL (39% of the database) were checked against the World Register of Marine Species (WoRMS; <http://www.marinespecies.org/>).
3. This left 52 genera absent from the COL and WoRMS, as well as 557 species names missing in COL and 47 species names missing in WoRMS. Missing species names under their genus in the COL were searched for in WoRMS.
4. We searched for all remaining species in other taxonomic databases. Global Biodiversity Information Facility (GBIF; <https://www.gbif.org/>) was prioritised, followed by Atlas of Living Australia (<https://bie.ala.org.au/>), Lichen Portal (<https://lichenportal.org/portal/>), Algae Base (<http://www.algaebase.org/>), Mycobank (<http://www.mycobank.org/>), Encyclopedia of Life (<https://eol.org/>), Tropicos (<http://www.tropicos.org>) and Wikispecies (<https://species.wikimedia.org/>).
5. Antarctic nematode experts updated some of the relevant taxonomic information for nematodes as online reference sources were not up to date (see Acknowledgments).
6. The remaining species that were not present in any taxonomic database were searched for in scientific publications and grey literature. Species with no locatable taxonomic record in any taxonomic database were removed from our database.
7. When the classification between databases differed, the classification with the most recent update (see above) was used. Care was taken to ensure the Taxon ID (Table 4) was updated when the genus or species was changed to match a record already present in the database. All changes and sources were recorded in the fields: “taxonRemarks” and “originalNameUsage” (Table 4).
8. The identifier of the taxonomic source database was recorded where available (see Table 4).
9. Whilst all care was taken to resolve as many inconsistencies as possible, it is recognized that there are still likely to be some taxonomic inconsistencies in the database. Many records still have a lack of consensus on final taxonomy, and it is not possible to rectify all of these. The database will continue to change with time to ultimately reflect modern understanding. This is particularly relevant considering we are in the midst of substantial taxonomic upheaval from the relatively new uptake of high throughput genetic sequence analysis.

**Database finalisation, validation, and cross-checking:** The following processes were undertaken to finalise the database:

1. Checked and added/edited dates to records missing collection/observation dates (from original sources – noting that many records only had the year they were collected and for 6297 records the collection/observation year could not be determined). Publication date (the date the record was published) was also included where possible (See also Figure 5 and Table 2 for more information on collection/observation date).
2. Added location names for vertebrates where available (transcribed directly from the ASPA Management Plans and joined location information for the penguin colonies from Lynch et al. (2013), Lynch & LaRue (2014), and the MAPPPD Penguin Database - Humphries et al. (2017).
3. In some cases, vertebrate colonies (particularly penguins) digitised from the ASPA Management Plans (Wauchope et al., 2019) were spread over multiple adjacent ice-free areas and were included in the database as multiple occurrences, even if the site was generally considered as a single colony. These multiple occurrences were combined into a single coordinate point where appropriate, and as following the colonies listed in MAPPPD (Humphries et al., 2017). This means that penguin occurrences in the database can now be considered separate colonies.
4. Assigned each record to an ACBR and ASPA, where relevant.
5. Assigned each record a functional grouping (‘PTM_ID’, Table 4) based on the functional groups provided in Lee et al. (2022) (where terrestrial species were categorised into taxonomic groups based on an expected similar response to threats and management; see Table 5.
6. Removed duplicates from the database (using the following fields: ‘year’, ‘month’, ‘Publish_YEAR’, ‘Publish_SOURCE’, ‘taxonID’, ‘decimalLongitude’, ‘decimalLatitude’, ‘individualCount’).
7. Checked Taxon IDs for duplicates and errors, including a cross-check through the Australian Antarctic Data Centre against their Antarctic Taxonomy list. Wherever possible, Taxon IDs were reconciled.
8. Updated or combined some fields to align with Darwin Core Standards (<https://dwc.tdwg.org/>). Unnecessary fields were deleted.
   1. **Project personnel:**

Principal Investigators: Aleks Terauds, Steven L. Chown, Jasmine R. Lee

Co-investigators: Ben Raymond, Hannah Wauchope, Peter Convey, Dana Bergstrom, Sharon Robinson

Technical support/Quality assurance/Quality Control: Claire Mason, Charlotte Patterson, Anton Van de Putte, David Watts

### Class III. Data set status and accessibility

1. Status
   1. Latest update: 14 July 2024
   2. Latest archive date: 24 July 2024
   3. Metadata status: 17 November 2023
   4. Data verification: See Class II B.3.
2. Accessibility
   1. Storage location and medium: Data are available as Supporting Information (Data S1) and are also archived through the Australian Antarctic Data Centre at <https://doi.org/10.4225/15/59100ba9157f7>. The dataset conforms to the CCBY Attribution License (<http://creativecommons.org/licenses/by/4.0/>).
   2. Contact person: Aleks Terauds, 203 Channel Highway Kingston, Tasmania Australia, 7007; [aleks.terauds@aad.gov.au](mailto:aleks.terauds@aad.gov.au): +61 3 6232 3339
   3. Proprietary restrictions:
      1. Release date: On publication
      2. Citation: Terauds, A., Lee, J.R., Wauchope, H., Raymond, B., Bergstrom, D.M., Convey, P., Mason, C., Patterson, C.R., Robinson, S.A, Van de Putte, A., Watts, D. and Chown, S.L. (2025) The Biodiversity of Ice-free Antarctica Database. *Ecology*.
   4. Costs: None

### Class IV. Data structural descriptors

1. Data set file
   1. Identity and Size
2. Biodiversity_IceFree_Antarctica.csv; 35,654 records, 48 fields, 15.3 MB; no compression.
   1. Header information: See Table 4
   2. Alphanumeric attributes: Mixed
   3. Special characters/fields: See Table 4
   4. Authentication procedures: NA
3. Variable information
   1. Variable identity: Table 4
   2. Variable definition: Table 4
   3. Units of measurement: Table 4
   4. Data type
      1. Storage type: Table 4
      2. List and definition of variable codes: Table 4
      3. Range for numeric values: Table 4
      4. Missing value codes: NA
      5. Precision: NA

**Table 2** Summary of the number of data records for most common genera (top 25 by number of records presented)

| Genus | Number of records | Date Range | Genus | Number of records | Date Range |
| --- | --- | --- | --- | --- | --- |
| *Pygoscelis* | 5109 | 1895 - 2019 | *Pohlia* | 317 | 1900 - 2008 |
| *Stercorarius* | 1492 | 1956 - 2017 | *Eudorylaimus* | 302 | 1901 - 2008 |
| *Larus* | 1014 | 1960 - 2017 | *Rhizocarpon* | 302 | 1934 - 2008 |
| *Buellia* | 892 | 1902 - 2009 | *Acarospora* | 300 | 1902 - 2009 |
| *Leucocarbo* | 871 | 1948 - 2017 | *Physcia* | 286 | 1902 - 2008 |
| *Bryum* | 870 | 1909 - 2010 | *Rhizoplaca* | 256 | 1911 - 2009 |
| *Usnea* | 773 | 1903 - 2008 | *Candelariella* | 255 | 1911 - 2008 |
| *Umbilicaria* | 667 | 1902 - 2008 | *Stereotydeus* | 253 | 1960 - 2009 |
| *Macronectes* | 665 | 1948 - 2017 | *Sanionia* | 252 | 1902 - 2005 |
| *Lecanora* | 653 | 1902 - 2009 | *Nanorchestes* | 245 | 1960 - 2008 |
| *Chionis* | 634 | 1965 - 2017 | *Cryptopygus* | 241 | 1903 - 2010 |
| *Sterna* | 615 | 1964 - 2017 | *Navicula* | 241 | 1957 - 2008 |
| *Caloplaca* | 589 | 1900 - 2010 | *Pagodroma* | 216 | 1960 - 2016 |
| *Xanthoria* | 534 | 1911 - 2008 | *Hypsibius* | 211 | 1958 - 2008 |
| *Syntrichia* | 405 | 1900 - 2005 | *Gomphiocephalus* | 198 | 1959 - 1998 |
| *Oceanites* | 402 | 1960 - 2017 | *Polytrichastrum* | 198 | 1900 - 2008 |
| *Ceratodon* | 400 | 1902 - 2008 | *Polytrichum* | 198 | 1900 - 2013 |
| *Lecidea* | 384 | 1911 - 2009 | *Prasiola* | 189 | 1940 - 2008 |
| *Schistidium* | 382 | 1903 - 2008 | *Friesea* | 188 | 1959 - 2010 |
| *Pseudephebe* | 380 | 1911 - 2008 | *Scottnema* | 188 | 1961 - 2009 |
| *Aptenodytes* | 353 | 1911 - 2018 | *Rinodina* | 181 | 1902 - 2008 |
| *Andreaea* | 352 | 1900 - 2008 | *Brachythecium* | 173 | 1900 - 2005 |
| *Plectus* | 341 | 1956 - 2008 | *Hennediella* | 173 | 1902 - 2008 |
| *Cladonia* | 335 | 1902 - 2002 | *Deschampsia* | 157 | 1940 - 2011 |
| *Daption* | 324 | 1948 - 2017 |  |  |  |

**Table 3.** Description of the broad categories of source data that were used to compile *The Biodiversity of Ice-free Antarctica Database*.

| datasetID | # records | Source Category | Description |
| --- | --- | --- | --- |
| ATD | 16,680 | SCAR Antarctic Biodiversity Database (subset) | The terrestrial subset of the original SCAR Biodiversity Database, consisting of various collections (see Table 1). Aleks Terauds is the original custodian of this subset. |
| ASPA | 3,291 | Antarctic Specially Protected Areas Database | A database containing terrestrial biodiversity records digitised from ASPA Management Plans (MP) between 2016 - 2018. This dataset was compiled by Hannah Wauchope and updated by Jasmine Lee*.  The coordinates for these records were either a) directly specified in the ASPA Management Plan, b) derived from the SCAR Gazetteer**, or c) derived from maps given in the ASPA Management Plan using Google Earth or Arc GIS***.  This dataset has been published as part of a scientific journal article (see Wauchope et al., 2019) and is hosted on the Australian Antarctic Data Centre (see <https://doi.org/doi:10.26179/5c1b15eedaeb6>).  *This dataset contains an updated version and additional records unique to this update can be identified in 'occurenceRemarks' with a "ASPA_v2" label (some duplicated records were also removed). ** SCAR Gazetteer available: <https://data.aad.gov.au/aadc/gaz/scar/>.  *** If coordinates were derived from maps using Google Earth or Arc GIS it is specified in 'occurenceRemarks'. |
| AD | 11,944 | Additional Data | Additional biodiversity records collected from the scientific literature by Jasmine Lee, Hannah Wauchope and Aleks Terauds between 2008 and 2019. The sources for these records are identified under 'Publish_SOURCE' and ‘associatedReferences’. Almost all of the 11,944 records in this category were sourced from peer reviewed publications, with only 6 records from unpublished sources. |
| MAPPPD | 3,739 | Penguin Map | Raw count data from MAPPPD (Mapping Application for Penguin Populations and Projected Dynamics) v. 3.0, downloaded in June 2021 from <http://www.penguinmap.com/mapppd>. Data covers emperor, chinstrap, Adelie and gentoo penguins. |

**Table 4.** Field names and associated details for *The Biodiversity of Ice-free Antarctica Database* (Biodiversity_IceFree_Antarctica.csv). Fields have been created to align, wherever possible, with the Darwin Core Standard (<https://dwc.tdwg.org>) and these are denoted by an *. In the database, NA’s were used when data were not available for a field, but some fields were not relevant to every occurrence and so were left blank.

| Field | Description | Additional Details | Type | Range |
| --- | --- | --- | --- | --- |
| occurrenceID* | Unique identifier for each record in database. |  | Integer | SATBD_1 - SATBD_35823 |
| taxonID* | Unique identifier for each species in database. | As per the Australian Antarctic Data Centres Biodiversity Database; https://data.aad.gov.au/aadc/biodiversity/. | Integer | 24 - 119186 |
| scientificName* | Scientific name of the species. | Genus and species combined in a single field (does not include infra specific information­) | Text | 2,304 unique values included |
| vernacularName* | Common name of the species. | Not relevant to all occurrence records (in which case the field was left blank). | Text | 48 unique values included |
| decimalLongitude* | Longitude of the record in decimal degrees (WGS 1984). | Records may have been snapped to the closest ice-free area (see "Snap_IFA"), thus "decimalLongitude" may not represent the original coordinates given in the source (see "originalLongitude"). | Numeric | -178.1326 - 173.84914 |
| decimalLatitude* | Latitude of the record in decimal degrees (WGS 1984). | Records may have been snapped to the closest ice-free area (see "Snap_IFA"), thus "decimalLatitude" may not represent the original coordinates given in the source (see "originalLatitude"). | Numeric | -86.64655 - -60.55068 |
| ACBR_ID | ID number of the Antarctic Conservation Biogeographic Region the record is located within. | See Terauds and Lee (2016); <https://onlinelibrary.wiley.com/doi/full/10.1111/ddi.12453>. | Integer | 1 - 16 |
| ASPA_ID | ID number of the Antarctic Specially Protected Area the record is located within. | See Wauchope et al. (2019) for the ASPA layer used; <https://www.nature.com/articles/s41467-019-08915-6>. | Integer | 101 - 175 |
| EventDate* | Date the record was collected; YYYY-MM-DD format. | ISO format 8601-1:2019. | Date | 1900-01-02 - 2019-01-25 |
| year* | Year the record was collected in. | If the source did not give a single or definitive year for the record and instead gave a period of multiple years, the first listed year was taken and additional information on the multiple seasons is available in "eventRemarks". | Integer | 1820 - 2019 |
| month* | Month the record was collected in. |  | Integer | 1 - 12 |
| day | Day the record was collected in. |  | Integer | 1 - 31 |
| Publish_YEAR | Year the record was published. |  | Integer | 1885 - 2021 |
| Publish_SOURCE | The name of the source of a record, typically a scientific article or an ASPA management plan. |  | Text |  |
| associatedReferences* | The full reference for literature associated with a record. |  | Text |  |
| basisOfRecord* | The type of record, whether it is cited from literature or an occurrence. |  | Text | Either ‘MaterialCitation’, ‘Occurrence’ or ‘PreservedSpecimen’ |
| kingdom* | The full scientific name of the kingdom the taxon is classified in. |  | Text | 8 unique values included |
| phylum* | The full scientific name of the phylum the taxon is classified in. |  | Text | 38 unique values included |
| class* | The full scientific name of the class the taxon is classified in. |  | Text | 96 unique values included |
| order* | The full scientific name of the order the taxon is classified in. |  | Text | 217 unique values included |
| family* | The full scientific name of the family the taxon is classified in. |  | Text | 410 unique values included |
| genus* | The full scientific name of the genus the taxon is classified in. |  | Text | 820 unique values included |
| specificEpithet* | The scientific species designation of the taxon. |  | Text | 1,611 unique values included |
| infraspecificEpithet* | Sub-species taxonomic delineation of the taxon. | Only included for occurrence records where relevant. | Text | 17 unique values included |
| nameAccordingTo* | The source in which the specific taxonomic hierarchy is defined. | All species except nematodes were resolved using online taxonomic databases (e.g., Catalogue of Life or WORMS). Most nematode species were resolved by nematode experts (see details in the “taxonRemarks” tab). | URL |  |
| scientificNameID* | The identifier of the species from the source in which the taxonomic hierarchy was defined. |  | Integer | 2497 - 1035621 |
| taxonRemarks* | Details of changes made to the records taxonomic hierarchy based on the original name of the record (which may have needed to be resolved to be compatible with currently accepted taxonomy). |  | Text |  |
| originalNameUsage* | The taxonomic name of the record as it appeared in the source (i.e., an earlier synonym of the currently accepted taxonomic name). |  | Text |  |
| originalNameTaxonID | The taxon ID of the record as per its original taxonomic name that appeared in the source. |  | Integer | 100168 - 119154 |
| alternateTaxonID | Taxon ID/s of taxonomic synonym/s of the species. |  | Integer | 59301 - 117740 |
| PTM_ID | Group ID number for the Priority Threat Management (PTM) group the species belongs to (See Lee et al., 2022) for details on PTM outputs). | Number identifier for PTM biodiversity groupings. Each PTM group includes species expected to respond in a similar way to threats and conservation management actions based on expert assessment. The PTM groups were determined by biodiversity experts in July 2017 as part of the Priority Threat Management workshop for terrestrial Antarctic biodiversity. Some species fall into multiple groups (e.g., some mosses), in which case they were labelled with both groups. If it was unclear which group a species should fall into, it was labelled as “*?*”. | Integer | 1 - 75 |
| PTM_NAME | Name of the PTM group the species belongs too. The name corresponds to the 'PTM_ID' number (Lee et al., 2022a). | Some species fall into multiple groups, in which case they were labelled with both groups. | Text | 93 unique values included |
| datasetID* | Original dataset in which the record was contained. | ATD - original Antarctic Terrestrial Database, ASPA - the ASPA database (see Wauchope et al.., 2019), AD – Additional Data collected from the primary scientific literature, MAPPPD, the Mapping Application for Penguin Populations and Projected Dynamics. See Table 3 for more details. | Text |  |
| institutionCode* | The institution or individual whom the record was collected by. | See also institution information in Table 1. | Text |  |
| collectionCode* | The code of the collection or dataset containing the record, as derived from the original Antarctic Terrestrial Database. |  | Integer | 4 - 92 |
| originalLongitude | Original longitude in decimal degrees of the record as given in, or derived from, its source. | The current longitude (see “decimalLongitude”) may have been ‘snapped’ to the closest ice-free area (see “Snap_IFA”). | Numeric | -178.1667 – 174.000 |
| originalLatitude | Original latitude in decimal degrees of the record as given in, or derived from, its source. | The current latitude (see “decimalLatitude”) may have been ‘snapped’ to the closest ice-free area (see “Snap_IFA”). | Numeric | -86.64571 - -60.5333 |
| Snap_IFA | Was the record snapped to the closest ice-free area (IFA), Y, N, or a name given. | Most biodiversity records from Antarctica are from ice-free areas, and if they do not appear to occur in one it is likely because either the original coordinates given were not highly accurate, or because the IFA layer is not perfect, hence they were snapped. The IFA layer used was the medium resolution rock outcrop layer (v7) from the Antarctic Digital Database (ADD; <https://www.add.scar.org/>).  Records with a N were already located within an IFA. Records with a Y were snapped to the closest IFA. Records with an alternative value given were not snapped to the closest IFA because they are in a known location that the ADD v7 rock-outcrop layer does not cover (e.g., ‘AdamIs’ is Adam Island). | Character |  |
| Dist_IFA | The distance (in m) to the closest IFA. If the record was snapped then this is the distance the record was moved to its new coordinates. |  | Integer | 0 – 411565.9 |
| coordinateUncertaintyInMetres* | Estimate of the accuracy of the record in m, where the value represents a diameter of uncertainty. | This largely applies to additional data records and ASPA records where the coordinates were derived from a location description in the source text. | Integer | 100 - 500000 |
| occurenceRemarks* | Comments about the occurrence record. | Comments may relate to how the coordinates were derived, related database IDs, status of the colony, or other information given in the source text.  For ASPA records: Coordinates that were derived from Google Earth or GIS, were normally derived given a map in the ASPA management plan the record was obtained from. ‘ASPA_v2’ records were not included in the ASPA database published with Wauchope et al. (2019). Further details on the ASPA dataset are given in the 'Database_Description' tab. Not relevant to all occurrence records (in which case the field was left blank). | Text |  |
| Location* | Named location of the record as given in the source text. | If no coordinates were given in the source text, this location name may have been used to derive coordinates from the SCAR Gazetteer (2013). Not relevant to all occurrence records (in which case the field was left blank). | Text |  |
| locationRemarks* | Comments about the location. | Comments may relate to additional location information, or may give information regarding how the coordinates were derived. Not relevant to all occurrence records (in which case the field was left blank). | Text |  |
| eventRemarks* | Comments about the occurrence event, typically indicating whether the observation year was derived from multiple listed observation years/seasons in the source. | Not relevant to all occurrence records (in which case the field was left blank). | Text |  |
| reproductiveCondition* | Information on the breeding status of the occurrence record (vertebrates only); B - breeding, NB - not breeding, U - unknown. | Not relevant to all occurrence records (in which case the field was left blank). | Text |  |
| dynamicProperties* | Further information on the occurrence relating to the residency status of the organism (i.e., resident of the area, or visitor). | Vagrant records are recognized here as 'Vagrant' (however, not all vagrant records are likely to have been recognized). Not relevant to all occurrence records (in which case the field was left blank). | Text |  |
| Num_Duplicates | The number of duplicate records that existed in the database (prior to being removed) for this record. | Duplication was based on all of the following fields being the same: taxonID, decimalLongitude, decimalLatitude, year, month, Publish_YEAR, Publish_SOURCE, individualCount. Duplication may have occurred if the same record was added into the database from different sources.  A value of '1' means there was only a single record (no duplicates detected). The number of duplicates were not calculated for additional data that was added in later versions of the database (e.g., the MAPPPD records). | Integer | 1 - 244 |

**** = as per Darwin Core Standard;*** [**https://dwc.tdwg.org/**](https://dwc.tdwg.org/)

**Table 5.** The groupings used to separate species into functional groups for use in the Priority Threat Management groupings of Lee et al., (2022a). Contained in the *PTM_ID* and *PTM_NAME* fields (see Table 4). Not every PTM group is represented within occurrences of *The Biodiversity of Ice-free Antarctica Database*, though all are included for consistency and in case of future additions to the database.

| PTM_ID | Category | Name | Example taxa from the biodiversity of ice-free Antarctica database and descriptions |
| --- | --- | --- | --- |
| 1 | Invertebrates | Midges | The two native Antarctic midges, i.e., *Belgica antarctica*, *Parochlus steinenii* |
| 2 |  | Freshwater nematodes, rotifers, tardigrades | Microfauna that survive in freshwater, e.g., nematodes - *Plectus* spp; rotifers - *Adineta* *grandis*, *Epiphanes* *senta*; tardigrades - *Acutuncus* *antarcticus*, *Diphascon* *langhovdense* |
| 3 |  | Terrestrial, wet soil nematodes, rotifers, tardigrades | Microfauna that primarily survive in wet soil, e.g., nematodes - *Plectus* spp., *Eudorylaimus* *antarcticus*, rotifers - *Adineta* *gracilis*; tardigrades - *Milnesium* *tardigradum*, *Hebesuncus* *ryani*, *Acutuncus* *antarcticus* |
| 4 |  | Terrestrial, moss associated nematodes, rotifers, tardigrades | Microfauna that are associated with mosses, e.g., nematodes - *Plectus* spp., *Eudorylaimus*; rotifers - *Adineta* *grandis*; tardigrades - *Acutuncus* *antarcticus*, *Echiniscus* *pseudowendti* |
| 5 |  | Entomobryomorpha springtails | e.g., *Isotoma* *klovstadi* |
| 6 |  | Poduromorpha springtails | e.g., *Gomphiocephalus* *hodgsoni* |
| 7 |  | Intertidal springtails | *Archisotoma brucei* |
| 8 |  | Intertidal mites |  |
| 9 |  | Terrestrial, free-living mites | All free-living mites, including some Cryptostigmata, Prostigmata, and Mesostigmata mites. Such as *Alaskozetes antarcticus, Coccorhagidia gressitti, Gamasellus racovitzai* |
| 10 |  | Intertidal enchytraeids and oligochaetes | e.g., *Christensenia* spp. |
| 11 |  | Terrestrial, dry soil nematodes | Nematodes that primarily survive in dry soil e.g., *Scottnema lindsayae* |
| 12 |  | Terrestrial, penguin rookery associated nematodes | Nematodes that primarily live in association with penguin rookeries, e.g., *Panagrolaimus* spp. |
| 13 | Vegetation | *Colobanthus quitensis* |  |
| 14 |  | *Deschampsia antarctica* |  |
| 15 |  | Bank-forming Mosses |  |
| 16 |  | Hydric Mosses |  |
| 17 |  | Mesic Mosses |  |
| 18 |  | Xeric Mosses |  |
| 19 |  | Truly aquatic mosses | i.e., Lake-dwelling moss |
| 20 |  | Leafy Liverwort | e.g., *Cephaloziella varians* |
| 21 |  | Freshwater or Limnetic algae |  |
| 22 |  | Fruticose & Foliose Lichens | e.g., *Usnea antarctica*, *Umbilicaria* spp. |
| 23 |  | Crustose Lichens | e.g., *Lecanora expectans*, *Xanthoria elegans* |
| 24 | Microbes | Mat-forming Terrestrial algae | e.g., *Prasiola crispa* |
| 25 |  | Biological soil crust communities |  |
| 26 |  | Dry soil microbial communities | Microbes that predominantly survive in dry soil |
| 27 |  | Lithic (endolithic, hypolithic and chasmolithic) microbial communities | Microbes that predominantly survive on/in rocks |
| 28 |  | Microbial mats, both lake and flush systems | Cyanobacteria |
| 29 |  | Wet soil microbial communities | Microbes that predominantly survive in the wet soil |
| 30 | Birds | Adélie penguins | Including monospecific ectoparasites for PTM analysis, e.g., lice *Lepidophthirus macrorhini* |
| 31 |  | Chinstrap penguins | Including monospecific ectoparasite for PTM analysis, e.g., lice *Lepidophthirus macrorhini* |
| 32 |  | Emperor penguins | Including monospecific ectoparasite for PTM analysis, e.g., lice *Lepidophthirus macrorhini* |
| 33 |  | Gentoo penguins | Including monospecific ectoparasite for PTM analysis, e.g., lice *Lepidophthirus macrorhini* |
| 34 |  | Antarctic shag | Including monospecific ectoparasite for PTM analysis, e.g., lice *Lepidophthirus macrorhini* |
| 35 |  | Greater sheathbill | Including monospecific ectoparasite for PTM analysis, e.g., lice *Lepidophthirus macrorhini* |
| 36 |  | Southern giant petrels | Including monospecific ectoparasite for PTM analysis, e.g., lice *Lepidophthirus macrorhini* |
| 37 |  | Skuas | South polar skuas, brown skuas, hybrids. Including monospecific ectoparasites for PTM analysis, e.g., lice *Lepidophthirus macrorhini* |
| 38 |  | Procellariids | Snow petrel, Southern Fulmar, Wilson's storm petrel, Antarctic petrels, Antarctic prion, Cape petrel; Including multispecific ectoparasites |
| 39* |  | Kelp gulls |  |
| 40* |  | Antarctic terns |  |
| 41* |  | Macaroni penguins |  |
| 42* |  | King penguins |  |
| 43* |  | Vagrants |  |
| 44* | Seals | Elephant seals |  |
| 45* |  | Fur seals |  |
| 46* |  | Weddell seals |  |
| 47* |  | Leopard seals |  |
| 48* |  | Crabeater seals |  |
| 49* |  | Ross seals |  |
| 50 | Other | Non-marine aquatic system plankton |  |
| 51 |  | Freshwater decapods |  |
| 52 |  | Freshwater copepods |  |
| 53 |  | Thalloid liverworts |  |
| 54* | Other | Invasive | Non-native species recorded in Antarctica |
| 56* |  | Monospecific ectoparasites |  |
| 57* |  | Multispecific ectoparasites |  |
| 58* |  | Mammal parasites |  |
| 59* |  | Snow algae |  |
| 61* |  | Other lake | Some single records of unusual groups from Antarctic lakes. Including Hydrozoa |
| 62* |  | Other intertidal | Some single records of other unusual intertidal species, including Antarctic limpet |
| 66* |  | Terrestrial protist | Terrestrial, heterotrophic free-living protists (e.g., in soils) |
| 67* |  | Terrestrial algae | For species that appear to be unicellular or do not appear to form mats |
| 68* |  | Sub-Antarctic beetles | Sub-Antarctic beetles |
| 69* |  | Arctic tern |  |
| 70* |  | Freshwater microbes | For microbes that do not appear to be in the soil. |
| 71* |  | Brackish microbes | Brackish water |
| 72* |  | Chromists | Simple multi-cellular species including diatoms and algae |
| 73* |  | Nematophagous fungi | Fungi that eat nematodes |
| 74* |  | Mushrooms | Mushroom forming fungi |
| 75* |  | Vegetative fungi | Fungi associated with moss/plants/lichens, including parasites/necrotrophs/biotrophs, etc. |

** = Not included in original PTM analyses, grouped later.*

- 1. Data format
     1. See Table 4

1. Data anomalies: See Class II. B.3.

### Class V. Supplemental descriptors

1. Data acquisition
   1. See Class II C.2. Data Collection
   2. Data entry verification procedures: See Class II C 3 Research Methods
2. Quality assurance/quality control procedures: See Class II C 3 Research Methods
3. Computer programs and data-processing algorithms: NA
4. Archiving
   1. Archival procedures: Data are archived at the Australian Antarctic Data Centre - doi:10.4225/15/59100ba9157f
   2. Redundant archival sites: NA
5. Publications and results: Iterations of this database have supported a range of studies, including continental biogeography (Terauds et al., 2012, Terauds and Lee 2016); protection of terrestrial biodiversity (Wauchope et al., 2019), Antarctic wilderness values (Leihy et al., 2019) and an Antarctic species inventory (Pertierra et al., 2024).
6. History of data set usage
   1. Data request history:
7. In addition to the above publications, the following requests to use these have been made:

2021:

Prof. David Keith, University of New South Wales (Australian Antarctic Science Project 4568 – An Ecosystem Typology of Antarctica).

2022:

Prof. Kerrie Wilson, Queensland University of Technology (Securing Antarctica’s Environmental Future, Theme 3 – Conservation Planning).

Prof. Steven Chown, Monash University (Securing Antarctica’s Environmental Future - Extension of type locality work, Phillips et al., 2022)

Prof Melodie McGeoch, Latrobe University (Securing Antarctica’s Environmental Future, Theme 2 - Biodiversity informatics).

Dr Luis Pertierra, University of Pretoria, “Frozen but lively: ecological research advances and knowledge gaps in terrestrial and freshwater Antarctic biodiversity science” (currently under review in *Science*).

**Acknowledgements**

We would like to acknowledge the support of the Scientific Committee for Antarctic Research (SCAR) largely through its Research Programs, including Regional Sensitivity to Climate Change (RiSCC), Evolution and Biodiversity in Antarctica (EBA), The State of the Antarctic Ecosystem (AntEco) and most recently through the Integrated Science to Support Antarctic and Southern Ocean Conservation (Ant-ICON). We would also like to acknowledge the original data custodians of the data extracted from the earlier SCAR Biodiversity Database (Table 1), and the numerous researchers who have collected the spatially explicit biodiversity data in ice-free Antarctica – without them this database would not have been possible. We would like to acknowledge the assistance of Johnathan Kool (AADC) in final checks and reconciliation of the taxonomy with the Australian Antarctic Data Centre and thank Ashley Shaw and the late Diana Wall and for assistance and advice on nematode taxonomy.

**References**

AADC. 2002. Antarctic Biodiversity Database, Version 1, Australian Antarctic Data Centre <https://data.aad.gov.au/metadata/records/antarctic_biodiversity_db>, accessed: 2023-02-09.

Araujo, R., V. V. Gupta, F. Reith, A. Bissett, P. Mele, and C. M. Franco. 2020. “Biogeography and Emerging Significance of Actinobacteria in Australia and Northern Antarctica soils”. *Soil Biology and Biochemistry* **146**: 107805. https://doi.org/ [10.1016/j.soilbio.2020.107805](https://doi.org/10.1016/j.soilbio.2020.107805).

Bánki, O., Roskov, Y., Döring, M., Ower, G., Hernández Robles, D. R., Plata Corredor, C. A., Stjernegaard Jeppesen, T., Örn, A., Vandepitte, L., Hobern, D., Schalk, P., DeWalt, R. E., Ma, K., Miller, J., Orrell, T., Aalbu, R., Abbott, J., Adlard, R., Aedo, C., et al. (2024). Catalogue of Life (Annual Checklist 2024). Catalogue of Life, Amsterdam, Netherlands. <https://doi.org/10.48580/dg9ld>

Brooks, S. T., J. Jabour, J. van den Hoff, and D. M. Bergstrom. 2019. “Our Footprint on Antarctica Competes with Nature for Rare Ice-Free Land”. *Nature Sustainability* **2**: 185-190. https://doi.org/10.1038/s41893-019-0237-y.

Burton-Johnson, A., M. Black, P. T. Fretwell, and J. Kaluza-Gilbert. 2016. “An Automated Methodology for Differentiating Rock from Snow, Clouds and Sea in Antarctica from Landsat 8 Imagery: A New Rock Outcrop Map and Area Estimation for the Entire Antarctic Continent”. *The Cryosphere* **10**: 1665-1677. https://doi.org/[10.5194/tc-10-1665-2016](https://doi.org/10.5194/tc-10-1665-2016).

Chan, Y., J.D. Van Nostrand, Zhou, S.B. Pointing and R.L. Farrell. 2013. “Functional ecology of an Antarctic dry valley”. *Proceedings of the National Academy of Sciences*, **110**: 8990-8995. <https://doi.org/10.1073/pnas.1300643110>.

Chown, S. L., and P. Convey. 2007. “Spatial and Temporal Variability Across Life's Hierarchies in the Terrestrial Antarctic”. *Philosophical Transactions of the Royal Society B: Biological Sciences* **362**: 2307-2331. https://doi.org/10.1098/rstb.2006.1949.

Chown, S. L., A. Clarke, C. I. Fraser, S. C. Cary, K. L. Moon, and M. A. McGeoch. 2015. “The Changing Form of Antarctic Biodiversity”. *Nature* **522**: 431-438. https://doi.org/ 10.1038/nature14505.

Chown, S. L., A. H. L. Huiskes, N. J. M. Gremmen, J. E. Lee, A. Terauds, K. Crosbie, Y. Frenot, K. A. Hughes, S. Imura, K. Kiefer, M. Lebouvier, B. Raymond, M. Tsujimoto, C. Ware, B. Van De Vijver, and D. M. Bergstrom. 2012. “Continent-Wide Risk Assessment for the Establishment of Nonindigenous Species in Antarctica”. *Proceedings of the National Academy of Sciences of the United States of America* **109**: 4938-4943. https://doi.org/10.1073/pnas.1119787109.

Convey, P**.**, E.M. Biersma, A. Casanova-Katny, and C.S. Maturana 2020. “Refuges of Antarctic Diversity”. Chapter 10 in: *Past Antarctica* (eds. Oliva, M. & Ruiz-Fernández, J.), pp. 181-200. Academic Press, Burlington, doi: 10.1016/B978-0-12-817925-3.00010-0.

Convey, P., S. L. Chown, A. Clarke, D. K. A. Barnes, S. Bokhorst, V. Cummings, H. W. Ducklow, F. Frati, T. G. A. Green, S. Gordon, H. J. Griffiths, C. Howard-Williams, A. H. L. Huiskes, J. Laybourn-Parry, W. B. Lyons, A. McMinn, S. A. Morley, L. S. Peck, A. Quesada, S. A. Robinson, S. Schiaparelli, and D. H. Wall. 2014. “The Spatial Structure of Antarctic Biodiversity”. *Ecological Monographs* **84**: 203-244. https://doi.org/10.1890/12-2216.1.

Convey, P., V. Bowman, S. L. Chown, J. E. Francis, C. I. Fraser, J. L. Smellie, B. Storey, and A. Terauds. 2018. “Ice Bound Antarctica: Biotic Consequences of the Shift from a Temperate to a Polar Climate”. In *Mountains, Climate and Biodiversity*, edited by C. Hoorn, A. Perrigo, and A. Antonelli, 355-373. New Jersey, USA: Wiley.

Convey, P. and L. S. Peck. 2019. “Antarctic Environmental Change and Biological Responses”. *Science Advances* **11**: eaaz0888. https://doi.org/10.1126/sciadv.aaz0888.

Deacon, G. E. R. 1955. “The Discovery Investigations in the Southern Ocean”. *Eos, Transactions American Geophysical Union* **36**: 877-880.

Dragone, N. B., M. A. Diaz, I. D. Hogg, W. B. Lyons, W. A. Jackson, D. H. Wall, B. J. Adams, and N. Fierer. 2021. “Exploring the Boundaries of Microbial Habitability in Soil”. *Journal of Geophysical Research: Biogeosciences*, **126**: e2020JG006052. https://doi.org/10.1029/2020JG006052.

Duffy, G. A., B. W. T. Coetzee, G. Latombe, A. H. Akerman, M. A. McGeoch, and S. L. Chown. 2017. “Barriers to Globally Invasive Species are Weakening Across the Antarctic”. *Diversity and Distributions* **23**: 982-996. https://doi.org/10.1029/2020JG006052.

Gerrish, L., P. Fretwell, and P. Cooper. 2020. “Medium resolution vector polygons of Antarctic rock outcrop (7.3) [Data set]. UK Polar Data Centre, Natural Environment Research Council, UK Research & Innovation. https://doi.org/10.5285/077e1f04-7068-4327-a4f2-71d863f70064.

Gressitt, J. L. 1961. “Problems in the Zoogeography of Pacific and Antarctic Insects”. *Pacific Insects Monograph* **2**: 1-94.

Hawes, I., C. Howard-Williams, Gilbert, K.A. Hughes, P. Convey and A. Quesada 2023. The need for increased protection of Antarctica’s inland waters. *Antarctic Science*. doi:10.1017/S0954102022000463

Hughes, K.A., Pescott, O., Peyton, J., Adriaens, T., Cottier-Cook, E., Key, G., Rabitsch, W., Tricarico, E., Barnes, D.K.A., Baxter, N., Belchier, M., Blake, D., Convey, P., Dawson, W., Frohlich, D., Gardiner, L., González-Moreno, P., James, R.,Malumphy, C., Martin, S., F. Martinou, A.F., Minchin, D., Monaco, A., Moore, N., Morley, S., Ross, F., Shanklin, J., Smith, K., Turvey, K., Vaughan, D., Vaux, A., Werenkraut, V., Winfield, I. & Roy, H. 2020. Invasive non-native species likely to threaten biodiversity and ecosystems in the Antarctic Peninsula region. *Global Change Biology* **26**, 2702-2716.

Hughes, K. A., Lowther, A., Gilbert, N., Waluda, C. M., & Lee, J. R. 2023. Communicating the best available science to inform Antarctic policy and management: A practical introduction for researchers. *Antarctic Science*, **35**, 438-472.

Hoskins, A. J., T. D. Harwood, C. Ware, K. J. Williams, J. J. Perry, N. Ota, J. R. Croft, D. K. Yeates, W. Jetz, M. Golebiewski, A. Purvis, T. Robertson, and S. Ferrier. 2020. “BILBI: Supporting Global Biodiversity Assessment Through High-Resolution Macroecological Modelling”. *Environmental Modelling & Software* **132**: 104806. <https://doi.org/10.1016/j.envsoft.2020.104806>.

Humphries, G. R. W., R. Naveen, M. Schwaller, C. Che-Castaldo, P. McDowall, M. Schrimph, and H. J. Lynch. 2017. “Mapping Application for Penguin Populations and Projected Dynamics (MAPPPD): Data and Tools for Dynamic Management and Decision Support”. *Polar Record* **53**(2): 160-166. <https://doi.org/10.1017/S0032247417000055>

Lee, C.K., D.C. Laughlin, E.M. Bottos, T. Caruso, K. Joy, J.E. Barrett, L. Brabyn, U.N. Nielsen, B.J. Adams, D.H. Wall. and D.W. Hopkins. 2019. “Biotic interactions are an unexpected yet critical control on the complexity of an abiotically driven polar ecosystem”. *Communications biology*, **2**:62 <https://doi.org/10.1038/s42003-018-0274-5>

Lee, J. R., A. Terauds, J. Carwardine, J. D. Shaw, R. A. Fuller, H. P. Possingham, S. L. Chown, P. Convey, N. Gilbert, K. A. Hughes, E. McIvor, S. A. Robinson, Y. Ropert-Couder, D. M. Bergstrom, E. M. Biersma, C. Christian, D. A. Cowan, Y. Frenot, S. Jenouvrier, L. Kelley, M. J. Lee, H. J. Lynch, B. Njåstad, A. Quesada, R. M. Roura, E. A. Shaw, D. Stanwell-Smit, M. Tsujimoto, D. H. Wall, A. Wilmotte, and I. Chadès. 2022a. “Threat Management Priorities for Conserving Antarctic Biodiversity”. *PLoS Biology* **20**. e3001921. https://doi.org/10.1371/journal.pbio.3001921.

Lee, J. R., M. J. Waterman, J. D. Shaw, D. M. Bergstrom, H. J. Lynch, D. H. Wall, and S. A. Robinson. 2022b. “Islands in the ice: Potential impacts of habitat transformation on Antarctic biodiversity”. Global Change Biology **28**:5865-5880. https://doi.org/10.1111/gcb.16331.

Lee, J. R., B. Raymond, T. J. Bracegirdle, I. Chadès, R. A. Fuller, J. D. Shaw, and A. Terauds. 2017. “Climate Change Drives Expansion of Antarctic Ice-Free Habitat”. *Nature* **547**: 49-54. https//doi.org/10.1038/nature22996.

Leihy, R. I., B. W. T. Coetzee, F. Morgan, B. Raymond, J. D. Shaw, A. Terauds, K. Bastmeijer, and S. L. Chown. 2020. “Antarctica’s Wilderness Fails to Capture Continent’s Biodiversity”. *Nature* **583**: 567-571. https://doi.org/10.1038/s41586-020-2506-3.

Lynch, H. J., R. Naveen, and P. Casanovas. 2013. “Antarctic Site Inventory Breeding Bird Survey Data, 1994–2013”. *Ecology* **94**: 2653-2653. https://doi.org/10.1890/13-1108.1.

Lynch, H. J., and M. A. LaRue. 2014. “First Global Census of the Adélie Penguin”. *The Auk* **131**: 457-466. <https://doi.org/10.1642/AUK-14-31.1>.

McCrea, R., R. King, L. Graham, and L. Börger. 2023. “Realising the Promise of Large Data and Complex Models”. *Methods in Ecology and Evolution* **14**(1): 4-11. https://doi.org/ 10.1111/2041-210X.14050.

Mawson, D. 1932. “The BANZ Antarctic Research Expedition, 1929-31”. *The Geographical Journal* **80**: 101-126.

O’Brien, K.M., E. L. Crockett, B. J. Adams, C. D. Amsler, H. J. Appiah-Madson, A. Collins, T. Desvignes, H. W. Detrich III, D. L. Distel, S. M. Eppley, and B. W. Frable. 2022. “The Time is Right for an Antarctic Biorepository Network”. *Proceedings of the National Academy of Sciences*, **119**(50): p.e2212800119. https://doi.org/10.1073/pnas.2212800119.

Ortiz, M., P. M. Leung, G. Shelley, T. Jirapanjawat, P. A. Nauer, M. W. Van Goethem, S. K. Bay, Z. F. Islam, K. Jordaan, S. Vikram, S. L. Chown, I. D. Hogg, T. P. Makhalanyane, R. Grinter, D. A. Cowan, and C. Greening. 2021. “Multiple Energy Sources and Metabolic Strategies Sustain Microbial Diversity in Antarctic Desert Soils”. *Proceedings of the National Academy of Science USA* **118**: e2025322118. <https://doi.org/10.1073/pnas.2025322118>.

Pacifici, M., Foden, W. B., Visconti, P., Watson, J. E., Butchart, S. H., Kovacs, K. M., Scheffers, B. R., Hole, D. G., Martin, T. G., Akçakaya, H. R., Corlett, R. T., Huntley, B., Bickford, D., Carr, J.A., Hoffmann, A. A., Midgley, G. F., Pearce-Kelly, P., Pearson, R. G., Williams, S. E., Willis, S.G., Young, B., Rondinini, C. (2015). “Assessing species vulnerability to climate change.” *Nature Climate Change*, 5(3), 215-224. <https://doi.org/10.1038/nclimate2448>

Patterson, C. R., K. J. Helmstedt, A. Terauds, and J. D. Shaw. In press. “A Multidimensional Assessment of Antarctic Terrestrial Biological Data”. *Diversity and Distributions.* Accepted 20^th^ June 2024.

Pertierra L.R., Varliero, G., Barbosa, A., Biersma, E.M., Convey, P., Chown, S.L., Cowan, D., De Los Rios, A., Escribano-Alvarez, P., Fontaneto, D., Fraser, C., Harris, M., Hughes, K., Griffiths, H., le Roux, P., Liu, X.P., Lynch, H., Majewska, R., Martinez, P.A., Molina-Montenegro, M., Olalla-Tarraga, M.A., Peck, L., Quesada, A., Ronquillo, C., Ropert-Coudert, Y., Sancho, L., Terauds, A., Vianna, J., Wilmotte, A., Hortal, J., Greve, M. 2024. “TerrANTALife 1.0 Biodiversity data checklist of known Antarctic terrestrial and freshwater life forms”. *Biodiversity Data Journal* 12: e106199. <https://doi.org/10.3897/BDJ.12.e106199>

R Core Team. 2019. “R: A Language and Environment for Statistical Computing” [Computer Software]. <https://www.R-project.org/>.

Shirihai, Н. 2008. “The Complete Guide to Antarctic Wildlife. Birds and Marine Mammals of the Antarctic Continent and the Southern Ocean”. Princeton University Press.

Terauds, A., S. L. Chown, F. Morgan, H. J. Peat, D. J. Watts, H. Keys, P. Convey, and D. M. Bergstrom. 2012. “Conservation Biogeography of the Antarctic”. *Diversity and Distributions* **18**: 726-741. <https://doi.org/10.1111/j.1472-4642.2012.00925.x>.

Terauds, A., and J. R. Lee. 2016. “Antarctic Biogeography Revisited: Updating the Antarctic Conservation Biogeographic Regions”. *Diversity and Distributions* **22**: 836-840. <https://doi.org/10.1111/j.1472-4642.2012.00925.x>.

Wauchope, H. S., J. D. Shaw, and A. Terauds. 2019. “A Snapshot of Biodiversity Protection in Antarctica”. *Nature Communications* **10**. **10**: 946. https://doi.org/10.1038/s41467-019-08915-6.

Wickham, H. 2007. “Reshaping Data with the Reshape Package”. *Journal of Statistical Software* **21(**12): http://www.jstatsoft.org/v21/i12/paper.
